# Dosimetric evaluation of cone‐beam CT‐based synthetic CTs in pediatric patients undergoing intensity‐modulated proton therapy

**DOI:** 10.1002/acm2.13604

**Published:** 2022-04-12

**Authors:** Khadija Sheikh, Dezhi Liu, Heng Li, Sahaja Acharya, Matthew M. Ladra, William T. Hrinivich

**Affiliations:** ^1^ Department of Radiation Oncology and Molecular Sciences Johns Hopkins University School of Medicine Baltimore Maryland USA

**Keywords:** adaptive radiotherapy, cone‐beam CT, image‐guided radiotherapy, intensity‐modulated proton therapy, pediatric cancer

## Abstract

**Purpose:**

To evaluate dosimetric changes detected using synthetic computed tomography (sCT) derived from online cone‐beam CTs (CBCT) in pediatric patients treated using intensity‐modulated proton therapy (IMPT).

**Methods:**

Ten pediatric patients undergoing IMPT and aligned daily using proton gantry‐mounted CBCT were identified for retrospective analysis with treated anatomical sites fully encompassed in the CBCT field of view. Dates were identified when the patient received both a CBCT and a quality assurance CT (qCT) for routine dosimetric evaluation. sCTs were generated based on a deformable registration between the initial plan CT (pCT) and CBCT. The clinical IMPT plans were re‐computed on the same day qCT and sCT, and dosimetric changes due to tissue change or response from the initial plan were computed using each image. Linear regression analysis was performed to determine the correlation between dosimetric changes detected using the qCT and the sCT. Gamma analysis was also used to compare the dose distributions computed on the qCT and sCT.

**Results:**

The correlation coefficients (*p*‐values) between qCTs and sCTs for changes detected in target coverage, overall maximum dose, and organ at risk dose were 0.97 (< .001), 0.84 (.002) and 0.91 (< .001), respectively. Mean ± SD gamma pass rates of the sCT‐based dose compared to the qCT‐based dose at 3%/3 mm, 3%/2 mm, and 2%/2 mm criteria were 96.5%±4.5%, 93.2%±6.3%, and 91.3%±7.8%, respectively. Pass rates tended to be lower for targets near lung.

**Conclusion:**

While insufficient for re‐planning, sCTs provide approximate dosimetry without administering additional imaging dose in pediatric patients undergoing IMPT. Dosimetric changes detected using sCTs are correlated with changes detected using clinically‐standard qCTs; however, residual differences in dosimetry remain a limitation. Further improvements in sCT image quality may both improve online dosimetric evaluation and reduce imaging dose for pediatric patients by reducing the need for routine qCTs.

## 1 INTRODUCTION

Proton therapy has the potential to reduce complications in pediatric cancers because of the reduction of integral dose to normal tissues when compared to photons.^1^ Proton therapy allows for conformal dose distributions with sharp dose falloffs as compared to intensity modulated photon therapy; however, inter‐ or intra‐fractional anatomical or physiological changes have the potential to cause geographical miss of the tumor and/or overdosing of critical structures.^2^ In particular, the finite range of protons in tissue depends on the radiological path length. A small positioning inaccuracy can result in changes in path length leading to large dosimetric variations at a point of interest. Conversely, photon dose distributions do not suffer from sensitivity to patient changes to the same degree. Frequent imaging and adaptive re‐planning can mitigate risks of significant changes in proton dosimetry caused by patient tissue changes.

Accordingly, verification or quality assurance CT (qCT) scans are often acquired using fan‐beam CT throughout the course of proton therapy, essentially repeating acquisition of the initial planning CT (pCT). qCTs allow anatomy to be evaluated and dose to be recomputed and directly compared to the pCT, but are time consuming for patients and lead to additional imaging dose. They are acquired either at a pre‐determined frequency or when daily or weekly on‐board imaging shows changes in normal tissues or tumor. The challenges associated with qCTs are important for pediatric patients who frequently require anesthesia to maintain a stable treatment position and may suffer increased deleterious effects from increased imaging dose.

In addition to qCTs, on‐treatment 3D imaging data are more frequently available due to the clinical usage of gantry‐mounted cone‐beam CT (CBCT) for patient alignment. The acquired CBCT images provide information on the position of bony anatomy, the gross volume changes, and the position of the patient on the treatment couch. Clinical proton dose calculations typically cannot be performed directly on CBCT images due to limitations in HU accuracy attributed to scatter and other artifacts, which are required to compute proton stopping power ratios and subsequent proton dose.^3^ Several groups^4^, ^5^, ^6^ have recently shown that CBCTs can be used for reliable proton dose calculation by utilizing deformable image registration (DIR) of the planning CT to the daily CBCT, yielding a “virtual” or “synthetic” CT. Synthetic CTs (sCTs) have been utilized in patients with head and neck cancer for adaptive planning.^4^, ^5^ Others have evaluated synthetic CT generation using daily CBCT of lung cancer patients treated using passive scattering proton therapy.^6^, ^7^ Limited work has evaluated the generation of sCTs in pediatric patients using on‐board CBCT for intensity‐modulated proton therapy (IMPT) dose calculations and evaluation. Reducing the number of qCTs these patients receive would allow for lower overall imaging dose and for those patients requiring anesthesia, significant reduction in anesthesia time.

In the present study, we used a ProBeat compact‐gantry pencil‐beam scanning proton therapy system (Hitachi, Tokyo JP) with a gantry‐mounted CBCT to evaluate dose using an automated sCT workflow in pediatric cancer patients. The addition of sCTs in our clinical workflow could allow clinicians to decide if a patient requires a qCT for adaptive planning, thus limiting the number of qCTs acquired over the course of treatment. To validate the method, we evaluated proton dose computed using the sCT approach in pediatric patients undergoing proton therapy, and assessed whether changes in dosimetry detected using standard qCTs were also detectable using sCTs. We hypothesize that the dose distribution between the qCT and sCT will not be significantly different, enabling similar detection of changes in dosimetry.

## 2 MATERIALS AND METHODS

### 2.1 Image acquisition and treatment planning

This retrospective study was approved by our institutional review board (IRB). Ten pediatric patients (age 0–18) treated at The Johns Hopkins Proton Center who underwent IMPT were included. Planning CTs were acquired for treatment simulation using a 64 slice Definition Edge Plus fan‐beam CT scanner (Siemens Healthineers, Erlangen DE) with 120 kV tube potential and 2 mm slice thickness with patients immobilized in the treatment position. Clinical IMPT plans were optimized using the RayStation 10A SP1 (RaySearch, Stockholm SE) treatment planning system (TPS) using robust optimization on the clinical target volume (CTV) accounting for 3.5% range uncertainty and setup uncertainty adjusted based on the treatment site and immobilization (ranging from 3 to 5 mm). Plans made use of 2–4 beams with or without a 4 cm range shifter depending on target depth. HU to mass density conversion was performed based on a stoichiometric calibration.^8^ Single field optimization (SFO) planning approaches were preferred but multi‐field optimization (MFO) was employed to achieve improved normal tissue sparing for some cases when deemed clinically beneficial. Final dose was computed using Monte Carlo with 0.5% statistical uncertainty. All plans were reviewed by the attending physician, physics, and underwent clinical peer review before final approval. The patient cohort included a variety of tumor sizes, locations, and anatomical changes that occurred throughout the treatment course listed in Table 1.

**TABLE 1:** Patient characteristics, treatment site, time from planning CT (pCT) to quality assurance CT (qCT), and gamma pass rates (γ) presented for each patient

Patient	Site	Δ*t* pCT–qCT (days)	γ_3%/3_ _mm_ (%)	γ_3%/2_ _mm_ (%)	γ_2%/2_ _mm_ (%)
A	Brain/C‐spine	17	100.0	99.4	99.0
B	Abdomen	20	96.9	94.8	90.9
C	Pelvis/Thigh	16	96.9	93.9	92.8
D	Pelvis	13	99.4	98.4	97.9
E	Pelvis	20	100.0	98.3	97.5
F	Brain	19	100.0	100.0	100.0
G	Mediastinum	27	87.2	81.7	77.2
H	Lung	20	89.9	85.1	80.4
I	Gluteus	20	98.7	90.7	89.3
J	Thigh	15	95.9	89.5	87.9
Mean±SD		18.9±4.0	96.5±4.5	93.2±6.3	91.3±7.8

**FIGURE 1:**
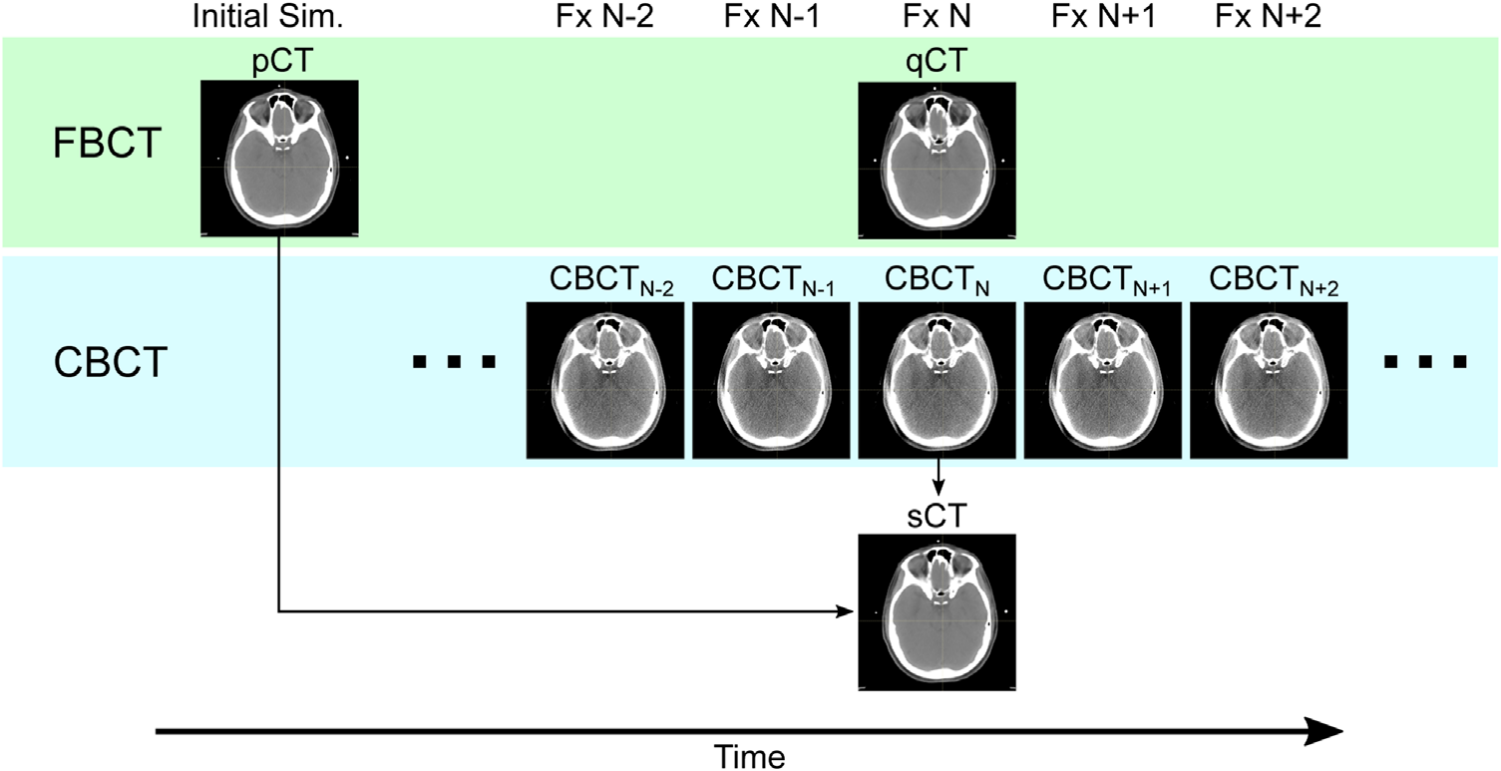
Flow chart indicating the timeline of image acquisition and analysis. The same fan‐beam CT (FBCT) was used to acquire the pCT and qCT. The CBCT used to generate the sCT and qCT used for comparison were acquired on the same day

The timeline for image acquisition is shown in Figure 1. For each patient in this study, a single pair of CBCT and qCT scans acquired on the same day mid‐treatment (fraction *N*) was selected for evaluation, thereby intending to capture the same patient anatomy and tissue changes that may have occurred since the original pCT was acquired. On‐treatment imaging included daily CBCT for verification of patient setup at the machine, and biweekly qCTs in the treatment position using the same fan‐beam CT scanner as the pCT for clinical dose calculation and plan evaluation. The proton gantry‐mounted CBCT system (Hitachi ProBeat, Hitachi Ltd) has a maximum field of view (FOV) of 38 cm. The images were acquired in full‐scan (179.9°–180°) or partial scan (45°–205°) mode at 100 kVp with resolutions ranging from 0.2–0.4 mm×0.2–0.4 mm×2–2.5 mm with an axial FOV of 20–37.5 cm and 69–145 number of slices depending on the anatomical site.

### 2.2 Synthetic CT generation

The CBCT was aligned to the pCT scan based on the image transformation extracted from the Patient Positioning Image and Analysis System (PIAS) at the time of treatment. Next, the pCT was deformed to the CBCT within the TPS using the built‐in anatomically constrained deformation algorithm (ANACONDA), which combines anatomical information and intensities, and was previously validated in lung cancer for CT‐to‐CT DIR and CT‐to‐CBCT DIR.^9^, ^10^, ^11^ We employed default DIR settings, which performed well based on manual review of the deformed images. The initial and final DIR grid resolution size were 5 mm isotropic and 2.5 mm isotropic, respectively. Initial and final Gaussian smoothing sigma were 2 and 0.33. Initial and final grid regularization weight was 400. Maximum number of iteration per resolution level was 1000.

Once the pCT was deformably registered to the CBCT, it was resampled in the CBCT frame of reference through an in‐house Python script, thus creating a synthetic CT (sCT). The contours were propagated from the pCT to the sCT using the DIR, were manually edited and reviewed by the attending physician. The original isocenter and treatment fields were transferred onto the sCT using the rigid alignment defined at time of treatment, and plan dose was re‐computed on the sCT using the Monte Carlo algorithm. With clinical implementation in mind, many of the analysis steps were automated using an in‐house Python script including image registration, re‐sampling, and contour propagation.

Similarly, the qCT acquired on the same day as the CBCT was rigidly registered to the pCT in the TPS using our standard clinical workflow. A DIR was performed to propagate contours from the pCT to the qCT using the same default approach, contours were manually adjusted and reviewed, and the treatment plan dose was re‐computed on the qCT.

### 2.3 Analysis

For each patient, the dose distributions were compared between the sCT and qCT using two approaches. First, 3D gamma analysis was performed comparing the sCT dose distribution to the qCT dose distribution. 3D gamma distributions were computed using the DoseComparison Module in 3D Slicer.^12^ Three criteria of the distance‐to‐agreement (DTA) and dose difference were evaluated (3%/3 mm, 3%/2 mm, 2%/2 mm).^13^ A 10% threshold of maximum dose was applied and gamma pass rates were computed for the region within the body contour.

Second, analysis was performed to determine whether the sCT was able to detect changes in dose distribution compared to the pCT similar to those detected by the qCT. For example, if the qCT shows a 4% decrease in target coverage compared to the pCT due to patient tissue change, we aim to assess whether the sCT shows the same 4% decrease in target coverage. To this end, we computed a variety of dose metrics *D* on the pCT, qCT, and sCT (denoted *D*_pCT_, *D*_qCT_, and *D*_sCT_). Dose metric change detected by the qCT (ΔqCT) and the sCT (textΔsCT) were computed as

(1) $$\Delta qCT\left(\%\right)\;=100*\left(D_{\mathrm{qCT}}-D_{\mathrm{pCT}}\right)/D_{\mathrm{pCT}}$$

(2) $$\Delta sCT\left(\%\right)\;=100*\left(D_{\mathrm{sCT}}-D_{\mathrm{pCT}}\right)/D_{\mathrm{pCT}}.$$

Three specific dose metrics were investigated including fractional volume of the CTV covered by 95% of the prescription dose (*V*95%), body maximum dose, and organ‐at‐risk (OAR) dose metrics. Two OAR dose metrics were analyzed for each patient, which were selected based on the specific treatment site. These included brainstem and optic chiasm maximum dose for patients with brain lesions, spinal canal maximum dose and pericardium mean doses for thoracic lesions, bladder and kidney mean doses for pelvis and abdominal lesions, and bone and skin maximum doses for extremities. Linear regression was performed between ΔsCT and ΔqCT in Python using scikit‐learn. Line of best fit, Pearson correlation coefficients and *p*‐values were computed to assess statistical significance of the correlations.

## 3 RESULTS

**FIGURE 2:**
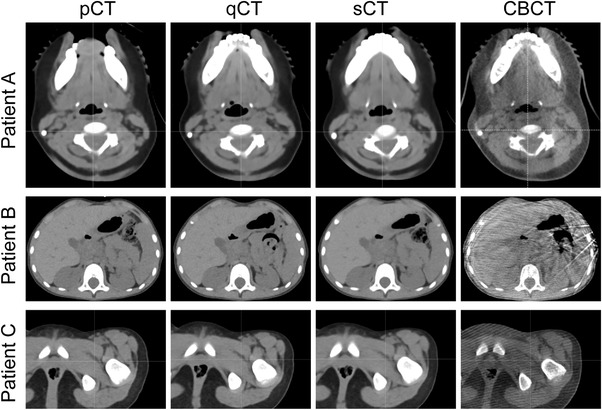
Example plan CT (pCT), quality assurance CT (qCT), synthetic CT (sCT), and cone‐beam CT from three example patients

Patient characteristics and treatment sites are shown in Table 1. The mean time between the pCT and qCT was 19 days. Figure 2 shows the pCT, qCT, sCT, and same day CBCT for three representative patients. The CBCT and sCT show similarities in mandible position for Patient A. This is also consistent with the same day qCT. The CBCT and sCT also show similarities in the airway, which may have changed between time of treatment and time of qCT acquisition. Similarities are also visually apparent in Patient B. Note that artifacts are present in the CBCT due to the boney anatomy. However, the sCT appears to preserve the gas volume as present on the CBCT despite the presence of artifacts.

**FIGURE 3:**
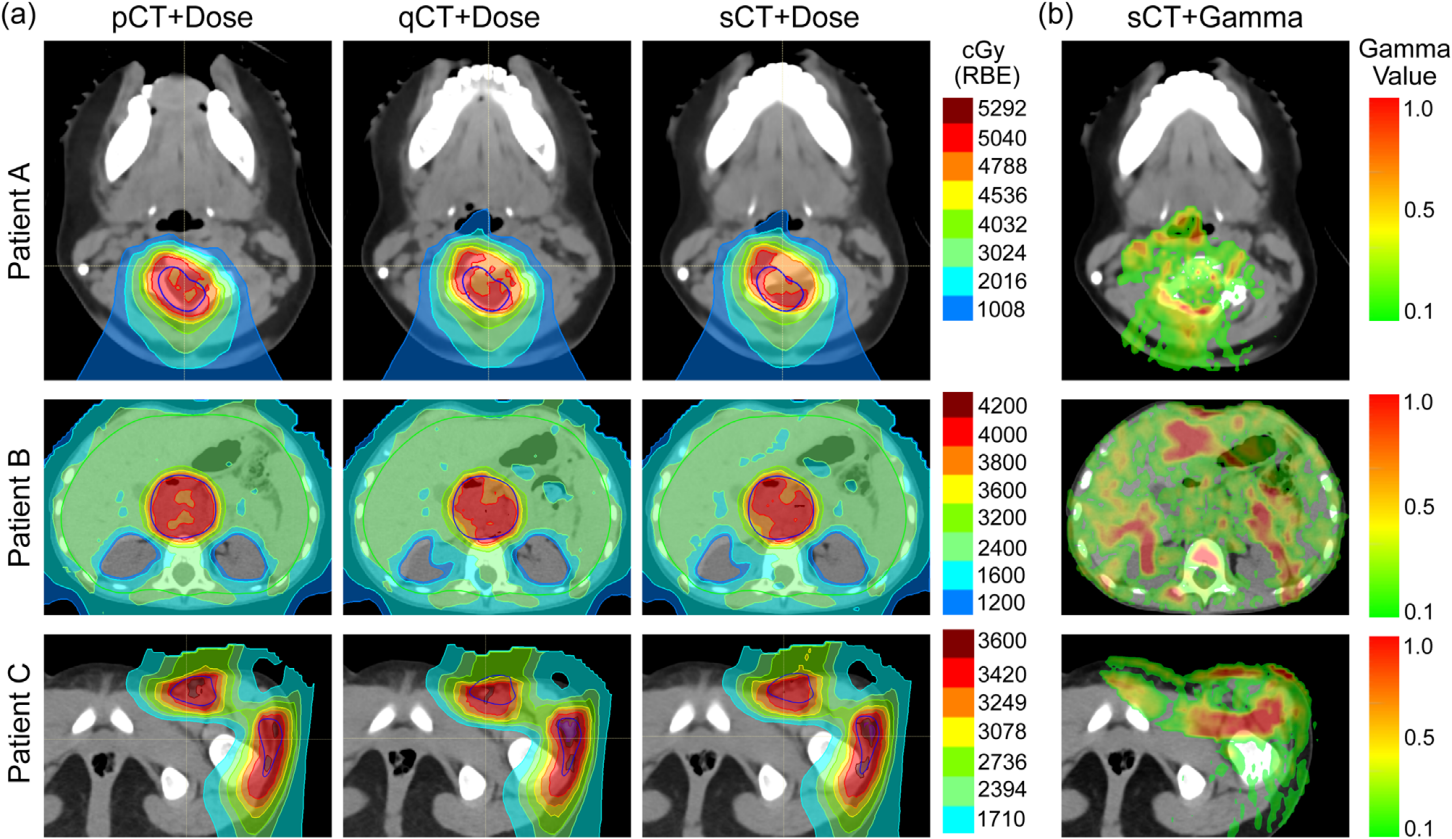
Example dose distributions and gamma analysis distributions for 3%2 mm criteria

Example distributions computed using the pCT, qCT, and sCT are shown in Figure 3. The CTVs are shown in navy blue. The gamma map (3%/2 mm) comparing the sCT and qCT is shown in the final column for each patient. Values of gamma < 1 are considered to pass. Cooler colors indicate a lower gamma value (i.e., more similar dose) and hotter colors indicate a higher gamma value (i.e., less similar dose). Values greater than 1 are considered to fail. It is apparent for Patient A that the lower dose in the airway is spatially similar between the qCT and sCT. The dose to the spinal canal is also similar between the qCT and sCT. This is reflected in the gamma map (pass rate: 99.4%). There are visibly apparent similarities between the qCT and sCT of Patient B specifically, the region around the kidneys. The target dose distribution is also similar. This is consistent with the gamma map demonstrating cooler green regions in the target area (pass rate: 94.8%). The reduction of gamma pass rate could also be attributed to the artifacts observed in the CBCT. The dose distributions computed on the qCT and sCT of Patient C demonstrate similarities in the high‐dose regions within the target. The dose discrepancies are observed near the anterior surface and anterior to the femoral head. This is consistent with the gamma map (pass rate: 93.9%).

Gamma analysis results for all patients are summarized in Table 1. Patients with lesions in the brain and pelvis had consistently high pass rates regardless of the gamma criteria used. Patients with lesions in the thorax and extremities had decreasing pass rates as the gamma criteria became tighter. Based on TG‐218, 7 out of 10 patients met the passing criteria for gamma analysis recommended for patient‐specific plan quality assurance (3%/2 mm ≥ 90%).^13^ Clinically, the passing rate is set to ≥ 95% at 3%/3 mm. Given this criteria, 8 out of 10 patients had acceptable dose differences. It is interesting to note the patients with lower passing scores had lesions in the thorax.

**FIGURE 4:**
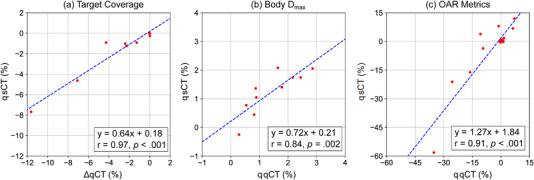
Scatter plots comparing dose metric changes detected using sCT (ΔsCT) to those detected using qCT (ΔqCT). Dose metric changes are computed relative to the original planning CT (pCT)

Figure 4 shows scatter plots of the change in dose computed using sCT (ΔsCT) versus the changes computed using the qCT (ΔqCT). A correlation coefficient and slope of 1 indicate that the sCT detects identical changes in dosimetry to the qCT. The correlation coefficients (*p*‐values) between qCTs and sCTs for changes detected in target coverage, overall maximum dose, and organ at risk dose were 0.97 (< .001), 0.84 (.002), and 0.91 (< .001), respectively. The slope of the line relating the changes in target coverage of 0.64 suggests that for every 1% change in coverage detected on the qCT there is a 0.64% change detected on the sCT (i.e., the sCT underestimates changes in target coverage compared to the qCT). Similarly, the slope of the line relating changes in body maximum dose of 0.72 suggests slight under‐estimation in change detected on the sCT compared to the qCT. However, the slope of the line relating changes in OAR metrics of 1.27 suggests that the sCT over‐estimates changes in OAR compared to the qCT.

## 4 DISCUSSION

In this study, we developed a sCT pipeline utilizing on‐board CBCT to evaluate IMPT dose distributions in pediatric cancer patients. We made the following observations: (1) daily CBCT acquired using the proton gantry‐mounted imaging system allows for the generation of sCTs, (2) the spatial dose distributions of the qCTs and sCT are similar when evaluated using gamma indices, and (3) changes in dose detected on qCT and sCT are correlated.

While there has been significant work focusing on the generation of sCT using MRI in pediatric patients^14^, ^15^ work focusing on CBCT‐based sCTs in pediatrics is limited. In this work, we aimed to utilize daily on‐board CBCTs acquired on a compact gantry Hitachi ProBeat system for this purpose. We generated CBCT‐based sCTs using an in‐house script within our TPS and computed proton dose. The use of CBCT to create a sCT by deforming the pCT has been demonstrated in several previous studies. In fact, recent work has utilized a 2D–3D DIR to generate sCTs in head and neck patients demonstrating an important need in the proton community and centers that do not have CBCT capabilities.^16^ Several groups have created virtual CBCTs by deforming the real CBCT to match the pCT.^6^, ^7^ These images were acquired on a gantry‐mounted CBCT system (Ion Beam Applications (IBA) for patients who had undergone passively scattered proton therapy. sCTs have been shown to be a potential surrogate for qCTs for proton treatment verification in the context of head and neck malignancies^4^, ^5^, ^17^; however, these retrospective studies used data from linear accelerator CBCT systems in patients undergoing photon radiotherapy. Other groups have generated sCTs simulating different nasal cavity fillings to evaluate plan robustness in patients treated with proton therapy.^18^ To our knowledge, this is the first feasibility study utilizing images acquired on the compact gantry Hitachi ProBeat system to generate sCT for patients undergoing IMPT.

It is important to note that the sCT represents the position the patient was in during treatment. This pipeline has the potential to be used for dose tracking so that daily dose can be recalculated using the TPS. The user is able to import the daily CBCT and corresponding image registration into the TPS. The time taken to import the image set and generate the sCT is approximately 10 min. Using an in‐house graphical user interface, the user is able to rigidly or deformably propagate the targets and OARs onto the sCT. Taken together, this pipeline has the potential to be used as a decision support tool for clinicians, where the visualization of daily deposited dose could lead to the initiation of a qCT, rather than relying on a pre‐determined frequency of qCTs.

The gamma index was used to evaluate the spatial dose differences between the qCT and sCT. Dose distributions calculated on the sCT showed good agreement to dose calculated on those of the qCT (average passing rates 96% per plan for 3%/3 mm). This is consistent with what has been reported in adult head and neck patients (average passing rate of 94% for 3%/3 mm) who were originally treated with photons and were retrospectively replanned for proton therapy.^19^ It is interesting to note that the sites with the lowest pass rates corresponded to the mediastinum (87%) and lung (90%). This may reflect the limitations of deformable registration, where the displacement between ribs and lung may not be accurately modeled without specialized algorithms.^20^ Indeed, the results of this study are dependent upon the performance and limitations of the DIR chosen. There is a large scope to continue developing DIR algorithms for site‐specific issues. It should be noted that similar to previous work,^5^ the time between pCT and time of treatment (qCT) was not correlated with the gamma pass rate (result not shown), potentially suggesting that this approach may be applied throughout a treatment course to monitor dosimetry.

Finally, we assessed the ability of the sCT to detect the same changes in dosimetry as the qCT by computing dose metrics on the pCT, qCT, and sCT. Our results suggest that while the sCT and qCT do not detect identical changes, the detected changes are strongly correlated such that a change detected on the sCT would be indicative of a similar change detected on the qCT. While these results preclude the ability to use the sCT for direct quantitation or plan adaptation, it may be a valid approach to identify patients who require a qCT for quantitative dose assessment and potential plan adaptation. We utilize dosimetic thresholds to determine the need for adaptive planning when analyzing the qCTs. These thresholds tend to be site (and case) specific, so general statements about specific threshold levels are beyond the scope of this report. It should be noted that we currently perform anywhere between 3–5 qCTs for pediatrics’ patients. A qCT is always performed on the first or second fraction. The anesthesia time including treatment and qCT varies from 35–60 min. If a patient undergoes five qCTs over the course of treatment while being under anesthesia for maximum 60 min, that is five additional hours of anesthesia. With the sCT approach described in the present study, a qCT is still required to generate an adaptive plan. In other words, the sCTs are valid only for dose evaluation but not planning. However, the sCTs could eliminate the need for most routine qCTs for eligible patients whereby the sCT is used to assess dose, and in instances when dosimetry has degraded, a conventional qCT may be acquired. For some patients, this workflow could eliminate most anesthesia time associated with routine qCTs. We are currently investigating clinical implementation details of this sCT workflow which will be reported separately.

The proposed sCT approach and overall study are subject to several important limitations, including the small patient cohort included in this preliminary study. We chose to use same‐day qCT as a ground truth for comparison in this study. While patient anatomy should be nearly identical between the sCT and same‐day qCT, differences in patient setup including overall body position, bowel, rectum, and bladder filling would introduce differences between the sCT and qCT. The sCT is computed based on the patient in the true treatment position as determined at the machine using CBCT. A difference in setup observed on the qCT may not be indicative of how the patient was treated, and must be carefully monitored and minimized through immobilization. A limitation of the sCT approach itself is the relatively small CBCT FOV. Since the proton dose calculation depends critically on accurately modeling all tissue in the beam path, any occlusion of tissue in the CBCT prevents the generation of a sCT for dose calculation. Indeed, this characteristic makes the sCT approach better suited to small pediatric patients who may be fully encompassed in the CBCT FOV. Furthermore, it should be noted that the quality of the sCT depends on the quality of the CBCT which may be impacted by the scan protocol and reconstruction technique.

## 5 CONCLUSIONS

Taken together, this work presents the generation of sCT using CBCTs acquired on a proton gantry‐mounted system in pediatric patients undergoing IMPT. When used for dose calculations, the method demonstrated strong agreement with the dose distribution computed on the same day verification qCTs that are used for clinical dose evaluation while patients are under treatment. Furthermore, target coverage and OAR doses between the sCT and qCTs were correlated suggesting that this method can be used for dose evaluation, potentially mitigating imaging dose and time required for pediatric patients treated using proton therapy.

## AUTHORS’ CONTRIBUTION

Khadija Sheikh was responsible for the data acquisition, analysis, and preparation of manuscript; Dezhi Liu was responsible for data analysis and final approval of the manuscript; Heng Li was responsible for data analysis and final approval of the manuscript; Sahaja Acharya was responsible for data analysis, interpretation of results, and final approval of the manuscript; Matthew M. Ladra was responsible for conceptualizing the work, data acquisition and final approval of the manuscript; William T. Hrinivich was responsible for conceptualizing the work, data acquisition, analysis, and interpretation of results.

## CONFLICT OF INTEREST

The authors declare that there is no conflict of interest that could be perceived as prejudicing the impartiality of the research reported.
